# *IRGM* rs13361189 polymorphism may contribute to susceptibility to Crohn’s disease: A meta-analysis

**DOI:** 10.3892/etm.2014.1736

**Published:** 2014-05-28

**Authors:** YAO LU, CHUN-YU LI, SHU-SEN LIN, PENG YUAN

**Affiliations:** Department of Anorectal, The Fourth Affiliated Hospital of China Medical University, Shenyang, Liaoning 110032, P.R. China

**Keywords:** IRGM, Crohn’s disease, polymorphism, meta-analysis

## Abstract

The aim of the present meta-analysis was to evaluate the correlation between a common polymorphism, rs13361189 C>T in the immunity-related GTPase M (*IRGM*) gene, and susceptibility to Crohn’s disease (CD). The PubMed, CISCOM, CINAHL, Web of Science, Google Scholar, EBSCO, Cochrane Library and CBM databases were investigated from database inception through to October 1, 2013 without the application of any language restrictions. The meta-analysis was performed using STATA 12.0 software and the relative risk (RR) with a 95% confidence interval (CI) was calculated. Seven case-control studies were included with a total of 3,093 CD patients and 3,227 healthy control subjects. The results of the meta-analysis revealed that the *IRGM* rs13361189 polymorphism correlates with an increased risk of CD (T allele versus C allele: RR=1.25 with 95% CI, 1.04–1.50; P=0.016 and CT + TT versus CC: RR=1.21 with 95% CI, 1.03–1.42; P=0.018). A subgroup analysis conducted using a genotyping method indicated that the *IRGM* rs13361189 polymorphism was correlated with an increased risk of CD in the TaqMan^®^ (T allele versus C allele: RR=1.32 with 95% CI, 1.01–1.73; P=0.042) and the polymerase chain reaction-restriction fragment length polymorphism subgroups (T allele versus C allele: RR=1.80 with 95% CI, 1.32–2.45; P<0.001 and CT + TT versus CC: RR=1.61 with 95% CI, 1.19–2.18; P=0.018). However, no correlation was observed in the direct sequencing subgroup (P>0.05). Further subgroup analysis by sample size demonstrated significant correlations between the *IRGM* rs13361189 polymorphism and an increased risk of CD in the large sample-size subgroup (T allele versus C allele: RR=1.46 with 95% CI, 1.26–1.68; P<0.001 and CT + TT versus CC: RR=1.40 with 95% CI, 1.21–1.62; P<0.001). However, no correlation was identified between the *IRGM* rs13361189 polymorphism and CD risk in the small sample-size subgroup (P>0.05). The present meta-analysis indicated that the *IRGM* rs13361189 polymorphism may contribute to susceptibility to CD. Thus, *IRGM* rs13361189 polymorphism may be a potential biomarker for the early diagnosis of CD.

## Introduction

Crohn’s disease (CD), also identical to Crohn syndrome or regional enteritis, refers to one form of inflammatory bowel disease that may affect any part of the gastrointestinal tract between the mouth and the anus, resulting in various symptoms (1). In recent years the incidence and prevalence rates of CD have rapidly increased, contributing significantly to the burden on the health care system and exhibiting high morbidity and mortality rates (2). The underlying pathogenesis of CD remains unclear, but may result from interactions between environmental, immunological and bacterial factors (3). Various countries and nationalities have demonstrated different incidence rates of CD, which cannot be explained by living habits or other risk factors, suggesting that genetic polymorphisms may be crucial in the development of CD (4,5). Recently, studies have indicated that polymorphism of the immunity-related GTPase M (*IRGM*) gene is associated with an increased CD risk (6,7).

The IRGM protein is an atypical member of the interferon-inducible GTPase family, which is characteristically induced by interferons and provides resistance to intracellular pathogens (8). The human *IRGM* gene is located on chromosome 5q33.1 and contains five exons (9,10). Previous studies have shown that IRGM may have a key function in the innate immune response by regulating autophagy formation in response to intracellular pathogens (11,12). Furthermore, certain studies have demonstrated that autophagy is a potential pathogenic mechanism in CD (13,14). Therefore, it was hypothesized that single nucleotide polymorphisms (SNPs) in the *IRGM* gene may be important in the development of CD (15,16). Certain studies have indicated that a common polymorphism, rs13361189 C>T, in the *IRGM* gene may increase the risk of CD (17,18); however, individually published studies provided inconclusive results (19,20). Therefore, in the present study a meta-analysis of all eligible case-control studies was conducted to evaluate the correlation between the *IRGM* rs13361189 polymorphism and susceptibility to CD.

## Methods

### Literature search

The PubMed, CISCOM, CINAHL, Web of Science, Google Scholar, EBSCO, Cochrane Library and CBM databases were searched from inception through to October 1, 2013 without the application of any language restrictions. The following keywords and medical subject headings were used: (‘SNP’ or ‘mutation’ or ‘genetic polymorphism’ or ‘variation’ or ‘polymorphism’ or ‘single nucleotide polymorphism’ or ‘variant’) and (‘Crohn’s disease’ or ‘CD’) and (‘human immunity-related GTPase M’ or ‘IRGM’). In addition, a manual search was performed to obtain other potential articles.

### Selection criteria

In the present meta-analysis, studies were included when the following criteria were met: i) The study design was a clinical cohort or case-control study; ii) the study concerned the correlation between the *IRGM* rs13361189 polymorphism and susceptibility to CD; iii) the patients conformed to the diagnostic criteria of CD; and iv) the study provided sufficient information regarding the frequency of the *IRGM* rs13361189 polymorphism. Studies that did not meet the inclusion criteria were excluded. The most recent or the largest sample-size study was included when the authors published several studies regarding the same subject matter.

### Data extraction

Using a standardized form, the relevant data were systematically extracted from all the included studies by two researchers. The standardized form included the following items: Language of publication, publication year of article, first author’s surname, geographical location, design of the study, sample size, country of origin of the subjects, allele frequencies, source of the samples, genotyping method of the SNPs and evidence of Hardy-Weinberg equilibrium (HWE) in the healthy control subjects.

### Quality assessment

The methodological quality of the included studies was evaluated according to the Newcastle-Ottawa Scale (NOS) (21). The NOS criteria comprised: i) Subject selection (scores, 0–4); ii) comparability of subjects (scores, 0–2); and iii) clinical outcomes (scores, 0–3). The NOS scores ranged between 0 and 9 and a score ≥7 indicated that a study was of good quality.

### Statistical analysis

The meta-analysis was performed using STATA 12.0 software (StataCorp, College Station, TX, USA). The relative risk (RR) and the 95% confidence intervals (CI) were estimated. The Z test was used to estimate the statistical significance of the RRs, and the power calculations were conducted using power and sample size calculations (22). Cochran’s Q test and the I^2^ test were used to evaluate potential heterogeneity between the studies (23). When the Q-test result was P<0.05 or the I^2^ test result was >50% this indicated significant heterogeneity and the random-effect model was conducted; otherwise, the fixed-effects model was used. Subgroup and meta-regression analyses were conducted to investigate the potential sources of heterogeneity. Sensitivity analysis was performed by omitting each study in turn to evaluate the influence of single studies on the overall estimation. Begg’s funnel plots and Egger’s linear regression test were conducted to identify any publication bias (24).

## Results

### Characteristics of the included studies

**Figure 1:**
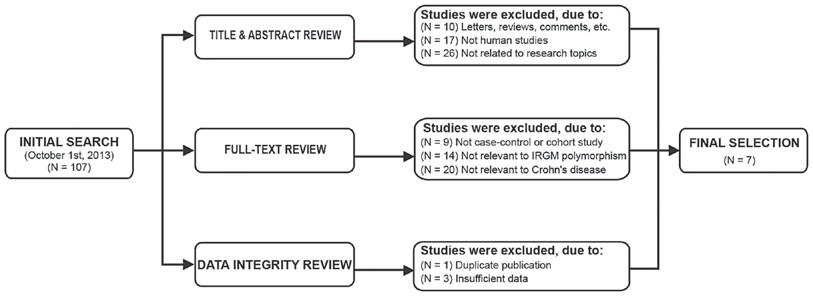
Flow chart of the literature search and study selection. Seven case-control studies were included in the present meta-analysis. IRGM, immunity-related GTPase M.

**Figure 2:**
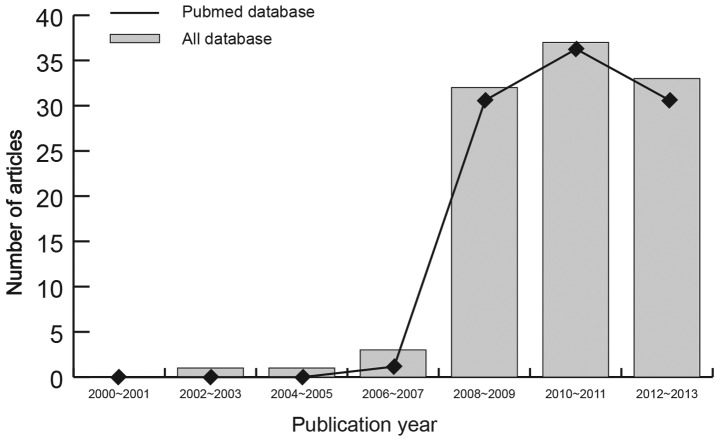
Distribution of the number of topic-related studies in the electronic databases.

A total of 107 articles were initially identified using the aforementioned keywords. The titles and abstracts of the articles were reviewed and 53 articles were subsequently excluded; the full texts and data integrity for the remaining articles were reviewed and a further 47 studies were excluded. Finally, seven case-control studies were included in the present meta-analysis (17–20,25–27), with publication years that ranged from 2008 to 2013. The selection process of the eligible studies is shown in Fig. 1. The distribution of the number of topic-related studies in the electronic databases during the last decade is demonstrated in Fig. 2. A total of 6,320 subjects were included in the meta-analysis, which included 3,093 CD patients and 3,227 healthy control subjects. The power values that were calculated for the sample size of the included studies were >0.70. Six studies were conducted with Caucasian populations, whereas only one study was performed with an Asian population. The TaqMan^®^ method was conducted in five studies and the other two studies used polymerase chain reaction-restriction fragment length polymorphism (PCR-RFLP) and direct sequencing methods, respectively. The genotype frequencies of the controls were all in HWE (P>0.05) and the NOS scores of the included studies were >5 (moderate-to-high quality). The study characteristics and methodological quality are summarized in Table I.

### Quantitative data synthesis

**Figure 3:**
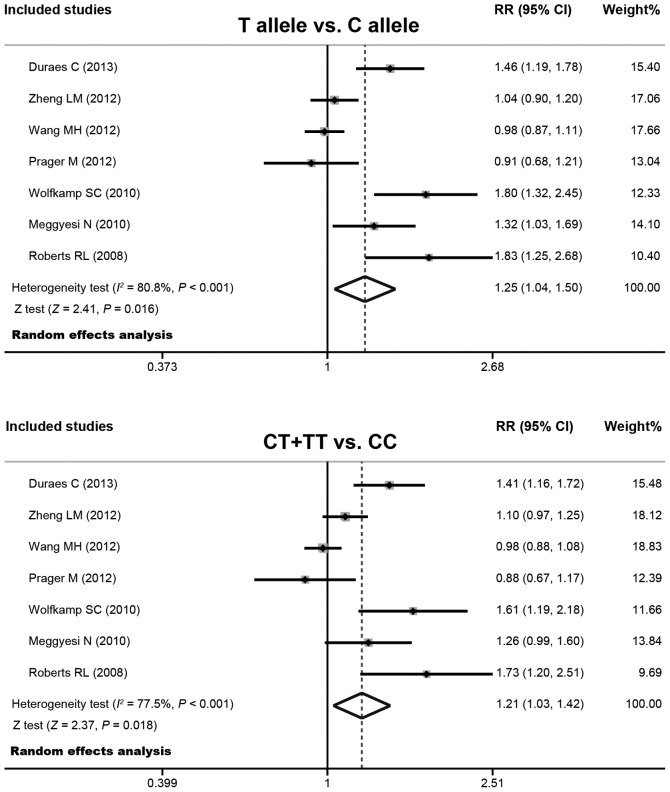
Forest plots for the association between the immunity-related GTPase M (*IRGM*) rs13361189 polymorphism and susceptibility to Crohn’s disease under the allele and dominant models. RR, relative risk; CI, confidence interval.

**Figure 4:**
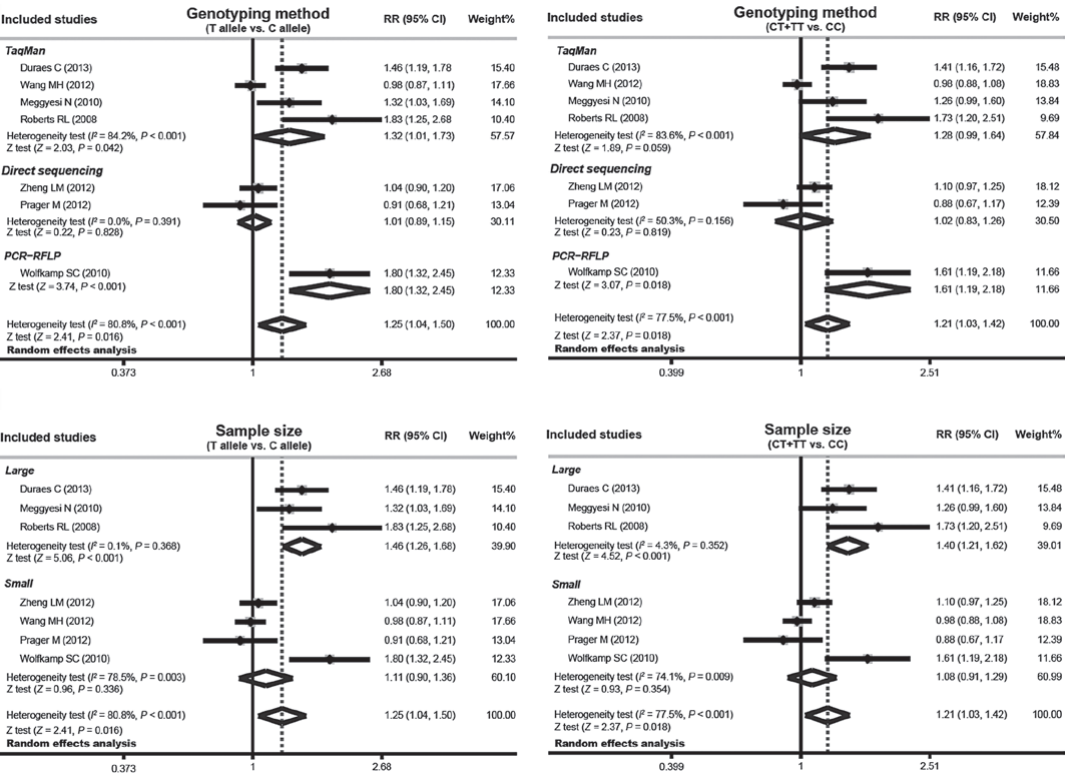
Subgroup analyses for the associations between the immunity-related GTPase M (*IRGM*) rs13361189 polymorphism and susceptibility to Crohn’s disease under the allele and dominant models. RR, relative risk; CI, confidence interval.

The random effects model was conducted due to the significant heterogeneity that existed between the studies. The present meta-analysis results revealed that the *IRGM* rs13361189 polymorphism correlates with an increased risk of CD (T allele versus C allele: RR=1.25 with 95% CI, 1.04–1.50; P=0.016 and CT + TT versus CC: RR=1.21 with 95% CI, 1.03–1.42; P=0.018; Fig. 3). The subgroup analysis that was conducted using the genotyping method indicated that the *IRGM* rs13361189 polymorphism was correlated with an increased risk of CD in the TaqMan (T allele versus C allele: RR=1.32 with 95% CI, 1.01–1.73; P=0.042) and PCR-RFLP subgroups (T allele versus C allele: RR=1.80 with 95% CI, 1.32–2.45; P<0.001 and CT + TT versus CC: RR=1.61 with 95% CI, 1.19–2.18; P=0.018), but not in the direct sequencing subgroup (P>0.05; Fig. 4). Further subgroup analysis by sample size demonstrated significant correlations between the *IRGM* rs13361189 polymorphism and an increased risk of CD in the large sample-size subgroup (T allele versus C allele: RR=1.46 with 95% CI, 1.26–1.68; P<0.001 and CT + TT versus CC: RR=1.40 with 95% CI, 1.21–1.62; P<0.001). However, no correlation was identified between the *IRGM* rs13361189 polymorphism and CD risk in the small sample-size subgroup (P>0.05).

**Figure 5:**
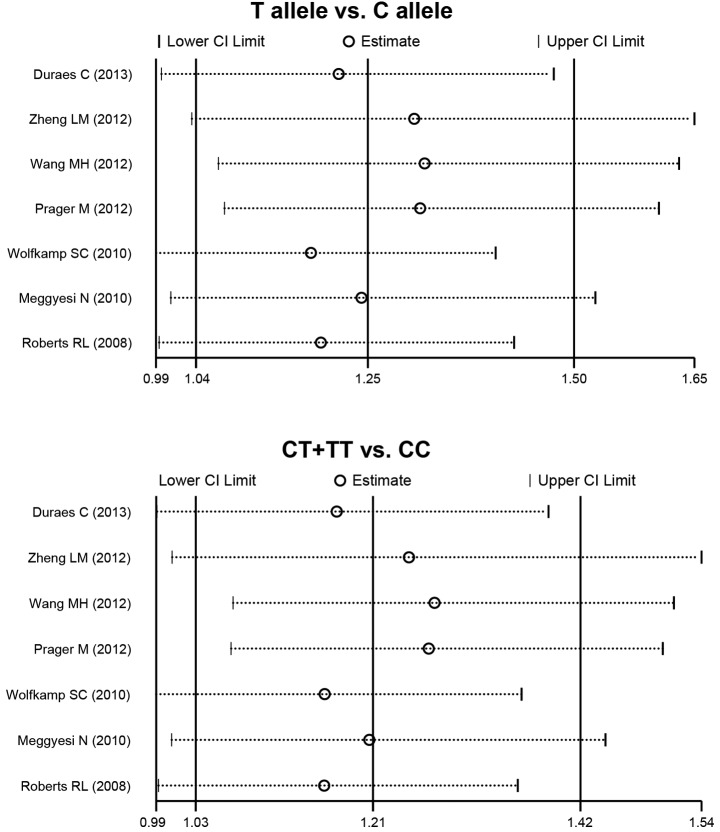
Sensitivity analysis of the summary odds ratio coefficients on the correlation between the immunity-related GTPase M (*IRGM*) rs13361189 polymorphism and susceptibility to Crohn’s disease under the allele and dominant models. The results were computed by omitting each study in turn. Meta-analysis random-effects estimates (exponential form) were used. The two ends of the dotted lines represent the 95% confidence interval (CI).

**Figure 6:**
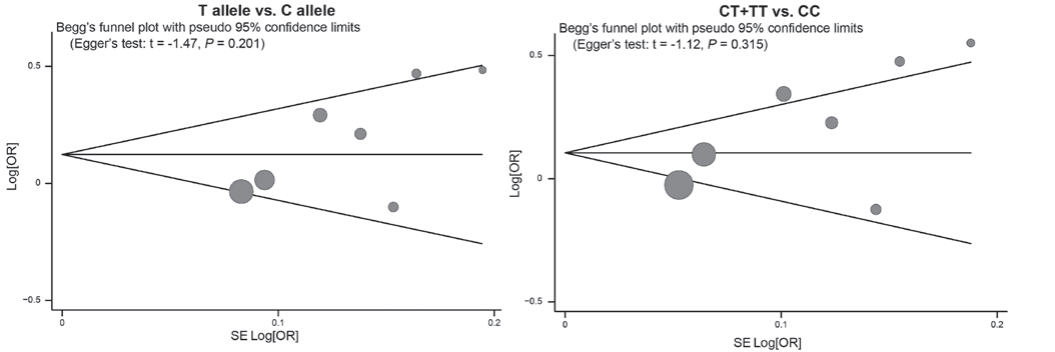
Begg’s funnel plot of publication bias regarding the correlation between the immunity-related GTPase M (*IRGM*) rs13361189 polymorphism and susceptibility to Crohn’s disease under the allele and dominant models. Each point represents a separate study for the indicated association and the horizontal line represents the mean magnitude of the effect. Log, natural logarithm; OR, odds ratio.

The results of the sensitivity analysis indicated that no single study influenced the overall pooled odds ratio (Fig. 5). Univariate and multivariate meta-regression analyses showed that sample size may be a predominant source of heterogeneity (P=0.003; Table II). There was no evidence of obvious asymmetry in the Begg’s funnel plots (Fig. 6) and the Egger’s test did not display strong statistical evidence for publication bias (T allele versus C allele: t=−1.47, P=0.201 and CT + TT versus CC: t=−1.12, P=0.315).

## Discussion

IRGM is responsible for the innate immune response via regulation of autophagy formation in response to intracellular pathogens (28). Cell homeostasis is dependent on the biosynthesis of macromolecules and the balance between catabolism and autophagy (29). In addition, autophagy is considered to be a key regulatory mechanism in the update, development and differentiation of cellular components and tissue remodeling (30). However, the interfered autophagy process may result in failure of the timely removal of injured cell structures, aging organelles, abandoned biological macromolecules and intracellular bacteria, which may trigger inappropriate immune responses, thus leading to the pathogenesis of chronic intestinal inflammation (31,32). Therefore, *IRGM* genetic polymorphisms that influence the normal expression of IRGM have been indicated to be associated with an increased risk of CD (7,16).

In the present meta-analysis, the correlation between the *IRGM* rs13361189 polymorphism and susceptibility to CD was evaluated. Seven independent case-control studies were included with a total of 3,093 CD patients and 3,227 healthy control subjects. The meta-analysis results demonstrated that the *IRGM* rs13361189 polymorphism correlates with an increased risk of CD, indicating that rs13361189 may be a causative factor for the incidence of CD. Although the exact function of the *IRGM* genetic polymorphism in CD is not fully understood, a potential explanation is that the *IRGM* genetic polymorphism may alter the function of IRGM, leading to the invasion of bacteria *in vivo*, resulting in tissue damage and chronic intestinal inflammation (33). As heterogeneity was clearly identified, stratified analyses based on the genotyping method and sample size were performed. The subgroup analysis using the genotyping method showed significant correlations between the *IRGM* rs13361189 polymorphism and an increased risk of CD in the TaqMan^®^ and the PCR-RFLP subgroups. However, no correlation was identified in the direct sequencing subgroup; this result was unreliable due to the small sample size. Further subgroup analysis by sample size showed a significant correlation between the *IRGM* rs13361189 polymorphism and an increased risk of CD in the subgroup with a large sample size. However, no correlation between the *IRGM* rs13361189 polymorphism and CD risk was identified in the subgroup with a small sample size. These results indicated that sample size may be a potential source of heterogeneity. The findings were predominantly consistent with previous studies, which demonstrated that the *IRGM* rs13361189 polymorphism may be strongly associated with the development and progression of CD, indicating that the *IRGM* rs13361189 polymorphism may be utilized as a biomarker for the early diagnosis of CD.

However, the present meta-analysis had certain limitations. Firstly, the results may not provide sufficient statistical power to estimate the correlations between the *IRGM* rs13361189 polymorphism and CD risk due to the relatively small sample size. Secondly, meta-analysis is a type of retrospective study that may lead to subject selection bias, thereby affecting the reliability of the results. Thirdly, the present meta-analysis failed to obtain the original data from the included studies, which may have further limited the evaluation of the potential roles of the *IRGM* genetic polymorphism in the development of CD. Furthermore, the inclusion criteria of the cases and controls were not well defined in all the included studies, which may have influenced the results.

In conclusion, the present meta-analysis indicated that the *IRGM* rs13361189 polymorphism may contribute to susceptibility to CD. Thus, the *IRGM* rs13361189 polymorphism may be a potential biomarker for the early diagnosis of CD. However, due to the abovementioned limitations, additional detailed studies are required to confirm these findings.

## Figures and Tables

**Table I:** Baseline characteristics and methodological quality of the included studies.

First author (Ref.)	Year	Country	Ethnicity	Sample size			Gender (M/F)		Age (years)		Genotyping method	HWE test (P-value)	NOS score
		
				Case	Control	Power	Case	Control	Case	Control			
Durães (25)	2013	Portugal	Caucasian	511	626	0.814	236/275	241/385	28.6±11.2	30.5 (9–83)	TaqMan	0.356	8
Zheng (19)	2012	China	Asian	318	318	0.764	154/164	156/162	37.2±11.4	36.7±12.3	Direct sequencing	0.142	8
Wang (27)	2012	USA	Caucasian	227	201	0.743	78/149	86/115	26.7±12.9	-	TaqMan	0.373	7
Prager (26)	2012	Germany	Caucasian	464	508	0.797	174/290	295/213	29.5±11.6	60.0±16.2	TaqMan	0.503	7
Wolfkamp (18)	2010	Netherlands	Caucasian	256	529	0.779	-	-	-	-	PCR-RFLP	0.239	6
Meggyesi (20)	2010	Hungary	Caucasian	810	469	0.828	434/376	251/218	37.1±12.6	40.5±11.5	TaqMan	0.743	8
Roberts (17)	2008	New Zealand	Caucasian	507	576	0.808	-	236/340	-	-	TaqMan	0.121	6

M, male; F, female; HWE, Hardy-Weinberg equilibrium; NOS, Newcastle-Ottawa Scale; PCR-RFLP, polymerase chain reaction-restriction fragment length polymorphism.

**Table II:** Univariate and multivariate meta-regression analysis of potential source of heterogeneity.

					95% CI	
					
Heterogeneity factor	Coefficient	SE	Z-value	P-value	LL	UL
Publication year						
Univariate	−0.589	0.039	−1.49	0.137	−0.136	0.019
Multivariate	−0.062	0.066	−0.94	0.348	−0.192	0.068
Genotyping method						
Univariate	0.053	0.150	0.35	0.724	−0.240	0.346
Multivariate	0.130	0.227	0.57	0.567	−0.315	0.575
Sample size						
Univariate	−0.258	0.140	−1.84	0.065	−0.532	0.016
Multivariate	−0.534	0.182	−2.94	0.003	−0.890	−0.177

SE, standard error; CI, confidence interval; UL, upper limit; LL, lower limit.

## References

[1] Frank DN, Robertson CE, Hamm CM (2011). Disease phenotype and genotype are associated with shifts in intestinal-associated microbiota in inflammatory bowel diseases. Inflamm Bowel Dis.

[2] Zheng JJ, Shi XH, Zhu XS (2011). A comparative study of incidence and prevalence of Crohn’s disease in mainland China in different periods. Zhonghua Nei Ke Za Zhi.

[3] Prescott NJ, Dominy KM, Kubo M (2010). Independent and population-specific association of risk variants at the IRGM locus with Crohn’s disease. Hum Mol Genet.

[4] Sivaram G, Tiwari SK, Bardia A (2012). Macrophage migration inhibitory factor, Toll-like receptor 4, and CD14 polymorphisms with altered expression levels in patients with ulcerative colitis. Hum Immunol.

[5] Joossens M, Van Steen K, Branche J (2010). Familial aggregation and antimicrobial response dose-dependently affect the risk for Crohn’s disease. Inflamm Bowel Dis.

[6] Brest P, Lapaquette P, Mograbi B (2011). Risk predisposition for Crohn disease: a ‘ménage à trois’ combining IRGM allele, miRNA and xenophagy. Autophagy.

[7] Glas J, Seiderer J, Bues S (2013). IRGM variants and susceptibility to inflammatory bowel disease in the German population. PLoS One.

[8] Taylor GA (2007). IRG proteins: key mediators of interferon-regulated host resistance to intracellular pathogens. Cell Microbiol.

[9] Moon CM, Shin DJ, Kim SW (2013). Associations between genetic variants in the IRGM gene and inflammatory bowel diseases in the Korean population. Inflamm Bowel Dis.

[10] Nord H (2010). Application of genomic and expression arrays for identification of new cancer genes. Dissertation.

[11] Mizushima N, Levine B, Cuervo AM, Klionsky DJ (2008). Autophagy fights disease through cellular self-digestion. Nature.

[12] Strausberg RL, Feingold EA, Grouse LH, Mammalian Gene Collection Program Team (2002). Generation and initial analysis of more than 15,000 full-length human and mouse cDNA sequences. Proc Natl Acad Sci U S A.

[13] Deretic V (2008). Autophagy, an immunologic magic bullet: Mycobacterium tuberculosis phagosome maturation block and how to bypass it. Future Microbiol.

[14] Eskelinen EL, Saftig P (2009). Autophagy: a lysosomal degradation pathway with a central role in health and disease. Biochim Biophys Acta.

[15] Xavier RJ, Huett A, Rioux JD (2008). Autophagy as an important process in gut homeostasis and Crohn’s disease pathogenesis. Gut.

[16] Palomino-Morales RJ, Oliver J, Gómez-García M (2009). Association of ATG16L1 and IRGM genes polymorphisms with inflammatory bowel disease: a meta-analysis approach. Genes Immun.

[17] Roberts RL, Hollis-Moffatt JE, Gearry RB (2008). Confirmation of association of IRGM and NCF4 with ileal Crohn’s disease in a population-based cohort. Genes Immun.

[18] Wolfkamp SC, Te Velde AA, Weersma RK (2010). Is there a role for Crohn’s disease-associated autophagy genes ATG16L1 and IRGM in formation of granulomas?. Eur J Gastroenterol Hepatol.

[19] Zheng LM, Pang Z (2012). Association of IRGM and ATG16L1 gene polymorphisms with Crohn’s disease in the Chinese Han population. Chin J Gastroenterol Hepatol.

[20] Meggyesi N, Kiss LS, Koszarska M (2010). NKX2–3 and IRGM variants are associated with disease susceptibility to IBD in Eastern European patients. World J Gastroenterol.

[21] Stang A (2010). Critical evaluation of the Newcastle-Ottawa scale for the assessment of the quality of nonrandomized studies in meta-analyses. Eur J Epidemiol.

[22] Dupont WD, Plummer WD (1990). Power and sample size calculations. A review and computer program. Control Clin Trials.

[23] Zintzaras E, Ioannidis JP (2005). HEGESMA: genome search meta-analysis and heterogeneity testing. Bioinformatics.

[24] Peters JL, Sutton AJ, Jones DR (2006). Comparison of two methods to detect publication bias in meta-analysis. JAMA.

[25] Durães C, Machado JC, Portela F (2013). Phenotype-genotype profiles in Crohn’s disease predicted by genetic markers in autophagy-related genes (GOIA study II). Inflamm Bowel Dis.

[26] Prager M, Büttner J, Haas V (2012). The JAK2 variant rs10758669 in Crohn’s disease: altering the intestinal barrier as one mechanism of action. Int J Colorectal Dis.

[27] Wang MH, Okazaki T, Kugathasan S (2012). Contribution of higher risk genes and European admixture to Crohn’s disease in African Americans. Inflamm Bowel Dis.

[28] Deretic V (2012). Autophagy as an innate immunity paradigm: expanding the scope and repertoire of pattern recognition receptors. Curr Opin Immunol.

[29] Folmes CD, Dzeja PP, Nelson TJ, Terzic A (2012). Metabolic plasticity in stem cell homeostasis and differentiation. Cell Stem Cell.

[30] He C, Klionsky DJ (2009). Regulation mechanisms and signaling pathways of autophagy. Annu Rev Genet.

[31] Caramés B, Taniguchi N, Otsuki S (2010). Autophagy is a protective mechanism in normal cartilage, and its aging-related loss is linked with cell death and osteoarthritis. Arthritis Rheum.

[32] Deretic V, Saitoh T, Akira S (2013). Autophagy in infection, inflammation and immunity. Nat Rev Immunol.

[33] McCarroll SA, Huett A, Kuballa P (2008). Deletion polymorphism upstream of IRGM associated with altered IRGM expression and Crohn’s disease. Nat Genet.

